# Scaling-up hepatitis C screening and treatment in Swiss outpatient psychiatric settings: A cost-effectiveness analysis

**DOI:** 10.1016/j.jhepr.2022.100464

**Published:** 2022-03-04

**Authors:** François Girardin, Alexandre Tuch, Lucy Eddowes, Martin Preisig, Francesco Negro

**Affiliations:** 1 Service of Clinical Pharmacology, Department of Laboratory Medicine and Pathology, Lausanne University Hospital (CHUV) and University of Lausanne, Lausanne, Switzerland; 2 Swiss Health Observatory [Obsan], Neuchâtel, Switzerland; 3 Costello Medical, London, UK; 4 Department of Psychiatry, Lausanne University Hospital (CHUV) and University of Lausanne, Lausanne, Switzerland; 5 Divisions of Gastroenterology and Hepatology and of Clinical Pathology, Geneva University Hospitals, Geneva, Switzerland

**Keywords:** Direct Antiviral Agents, Hepatitis C Infection, Screening Strategy, Cost-Effectiveness Model, Psychiatric Outpatients

To the Editor:

People with mental disorders (PMD) exhibit risk factors that increase their susceptibility to infectious agents such as HCV compared with the general population.^1^^,^^2^ Substance misuse is the most important predisposing factor for HCV infection; around 30–50% of PMD in Switzerland have a substance misuse disorder.^3^ The Swiss Federal Office of Public Health follows a risk-based HCV screening approach focussed on PMD with a substance misuse diagnosis. In previously published work using data from the University Hospitals of Geneva (HUG), Switzerland, we found that generalised screening and treatment for all inpatients admitted to psychiatric settings was cost-effective.[4], [5], [6] Since psychiatric care is transitioning towards intensive outpatient settings, we have now extended our methods to determine the cost-effectiveness over a lifetime time-horizon of a generalised approach *vs*. risk-based screening in both psychiatric inpatients and outpatients, where cost-effective screening may increase linkage-to-care and treatment initiation rates can be facilitated by direct-acting antivirals.^7^

The eligible population size (n = 160,796) was derived from health insurance data (number of psychiatric consultations; average number of consultations per person) and the adult population size of Switzerland,^8^^,^^9^ assuming that inpatients also receive outpatient care before or after their admission. Two scenarios for the epidemiological inputs for HCV prevalence and the proportion of people with a substance misuse disorder were defined using data from the Swiss Federal Statistical Office, analysed by the Swiss Health Observatory.

For Scenario I, inputs were derived from inpatients with a primary or secondary diagnosis of mental and behavioural disorder (ICD-10 codes F00-F99) aged 18-75 years (n = 125,564; 49.8% male; average age 51.65 years). The proportion with substance misuse diagnoses due to use of opioids (F11), cocaine (F14) and other psychoactive substances (F19) was 8.15%. HCV prevalence in this population was 6.43% and 0.24% in those without a substance misuse diagnosis. For Scenario II, in which only those admitted to a psychiatry setting were included (n = 49,015; 50.3% male; average age 43.96 years; 12.65% with substance misuse diagnoses), HCV prevalence was 4.98% in those with a substance misuse disorder and 0.21% in those without. The HCV prevalence estimates derived from recorded diagnoses are lower than the previous study, likely because the overall HCV prevalence in Switzerland is lower than in international areas such as Geneva (>40% foreign population).^10^^,^^11^ Further, cases may not have been recorded or identified in our analysis of diagnoses data. Therefore, the HCV prevalence estimates may be underestimated, enabling a conservative cost-effectiveness analysis.

We found that generalised HCV screening and treatment in Swiss inpatient and outpatient psychiatry settings was cost-effective, with an incremental cost-effectiveness ratio (ICER) of $31,447/quality-adjusted life-year (QALY) for Scenario I and $34,861/QALY for Scenario II, below the assumed willingness-to-pay threshold of $100,000 per QALY gained (Table 1). This was associated with incremental costs of $41,083,260 for Scenario I and $37,897,237 for Scenario II, giving a positive net monetary benefit of $89,557,614 for Scenario I and $70,813,109 for Scenario II. A generalised screening approach led to more patients initiating treatment, with 300 (Scenario I) and 249 (Scenario II) more patients linked to care and 250 (Scenario I) and 208 (Scenario II) more initiating treatment.

**Table 1:** **Summary of model results**.

	Scenario I (HCV prevalence based on those admitted to any setting)		Scenario II (HCV prevalence based on psychiatric setting admissions only)	
	Generalised screening approach	Current risk-based screening approach	Generalised screening approach	Current risk-based screening approach
No. of patients linked to care	1,012	712	1,106	856
No. of patients initiating treatment	844	594	922	714
Total costs	$96,452,779	$55,369,520	$101,301,408	$63,404,171
Total QALYs	140,856	139,550	142,966	141,879
Cost per person	$599.85	$344.35	$630.00	$394.31
QALYs per person	0.876	0.868	0.889	0.882
Incremental cost of generalised screening approach	$41,083,260		$37,897,237	
Incremental QALYs gained from generalised screening approach	1,306		1,087	
Incremental cost of generalised screening approach per person	$255.50		$235.69	
Incremental QALYs gained from generalised screening approach per person	0.01		0.01	
ICER	$31,447/QALY		$34,861/QALY	

ICER, incremental cost-effectiveness ratio; QALY, quality-adjusted life-year.

Deterministic sensitivity analyses found that the key inputs driving cost-effectiveness in both scenarios were the QALY gains associated with HCV treatment or being left untreated and the HCV prevalence within the population not currently screened. Probabilistic sensitivity analysis showed that the model outputs were robust: the probability of generalised screening being cost-effective was 97.1% for Scenario I and 96.3% for Scenario II. The probabilistic ICER was $32,242/QALY Scenario I and $34,633/QALY for Scenario II. These findings suggest that generalised HCV screening in the psychiatric setting is cost-effective compared to current risk-based screening of only patients with a substance misuse disorder.

Technology has reshaped the delivery of mental health services in recent years as increasing pressure to reduce care costs has led many healthcare providers to evolve from inpatient to more affordable and efficient outpatient care. Since psychiatric patients are more susceptible to infections such as HCV than the general population,^2^^,^^12^ rigorous screening of both inpatients and outpatients could lead to better identification of cases and timely linkage-to-care. We demonstrated that following a generalised screening approach targeting all inpatients and outpatients in Swiss psychiatric settings is likely to be cost-effective compared with the current risk-based approach. However, our assumption that the epidemiological inputs for the outpatient population are similar to the inpatient population could be considered a limitation. Furthermore, inputs extrapolated from the HUG data might not be representative of the overall Swiss psychiatric setting, potentially due to differences in patient behaviour and clinical protocols. Nevertheless, we explored variation in these inputs through sensitivity analyses. Overall, our results support the extension of HCV screening and treatment to the entire psychiatric setting irrespective of a substance misuse diagnosis, further supporting the World Health Organization’s goal to eliminate HCV by 2030.^13^

## Financial support

This work was supported by 10.13039/100016016Gilead and the 10.13039/501100006390University of Lausanne. The development of the model was funded by 10.13039/100016016Gilead. The funding sources did not have a role in the collection, analysis and interpretation of data; in the writing of the report; or in the decision to submit the article for publication.

## Authors’ contributions

François Girardin: conceptualisation, formal analysis, investigation, writing – review & editing. Alexandre Tuch: formal analysis, investigation. Lucy Eddowes: formal analysis, methodology, writing – original draft, writing – review & editing. Martin Preisig: writing – review & editing. Francesco Negro: writing – review & editing.

## Conflicts of interest

François Girardin is the representative of Swiss hospitals at the Federal Drug Commission (Federal Office of Public Health) and expert for InnoSuisse - the Federal Agency for Innovation (section Life Sciences). Francesco Negro advises Gilead, Merck and AbbVie, and has received a research grant from Gilead. Lucy Eddowes is an employee of Costello Medical.

Please refer to the accompanying ICMJE disclosure forms for further details.
